# Impact of culture-based bacteriological examination on diagnosis and treatment in cats with chronic nasal disease — Insights from a case series of 25 cats

**DOI:** 10.3389/fvets.2025.1687083

**Published:** 2025-10-31

**Authors:** Christin Emming, Jutta Verspohl, Andreas Beineke, Sarah Rösch

**Affiliations:** ^1^Small Animal Clinic, University of Veterinary Medicine, Hannover, Foundation, Hannover, Germany; ^2^Institute for Microbiology, University of Veterinary Medicine, Hannover, Foundation, Hannover, Germany; ^3^Department of Pathology, University of Veterinary Medicine, Hannover, Foundation, Hannover, Germany

**Keywords:** chronic rhinosinusitis, mycotic rhinitis, nasal discharge, nasal neoplasia, rhinoscopy

## Abstract

**Introduction:**

Diagnosing feline nasal cavity diseases typically involves computed tomography, rhinoscopy, mycological examination, and histopathology. Culture-based bacteriological examination (cBE) is frequently performed, though its diagnostic and therapeutic relevance remains uncertain. Chronic rhinosinusitis (CRS), a diagnosis of exclusion, often responds poorly to standard antibiotics. This retrospective case series aimed to describe the correlation of cBE findings (1) across nasal diseases and (2) treatment responses in CRS cases.

**Methods:**

Medical records of 25 cats with confirmed nasal disease using comprehensive diagnostics were reviewed.

**Results:**

Included were 11 CRS cats, 7 with mycotic rhinitis, and 7 with nasal neoplasia. In 24/25 cats, cBE was positive, with similar bacterial isolates across all groups. In CRS cats, treatment response did not consistently correlate with cBE results or antimicrobial susceptibility. 5/11 CRS cats showed clinical improvement following a 21-day doxycycline course. The remaining 6/11 CRS cats had not responded to previous targeted antibiotic therapy or empirical doxycycline for potential *Mycoplasma* species infection. 3/6 cats responded only to immunosuppressive therapy notably cyclosporine in two CRS cats, representing the first report in feline medicine. Non-responders were 3/6 CRS cats with marked turbinate destruction; 2/3 tested positive for feline herpesvirus 1.

**Conclusion:**

For diagnosing nasal diseases, cBE showed limited diagnostic relevance. In CRS, observations suggest that cBE may have limited diagnostic and therapeutic utility, leading to a clinical dilemma in interpretation. Empirical doxycycline treatment and immunosuppressive strategies, including cyclosporine, may be beneficial in selected CRS cases. Given the limitations of cBE, PCR testing for *Mycoplasma* species and viral pathogens may improve clinical decision-making in cats with CRS, both by helping to identify potential candidates for doxycycline treatment, and by assessing the risk of viral reactivation prior to initiating immunosuppressive therapy.

## Introduction

Feline upper respiratory tract disease (URTD) can be classified as either acute (duration of clinical signs ≤ 10 days) or chronic (duration of clinical signs > 10 days) (1–3). Acute URTD is commonly of infectious etiology, typically involving feline herpesvirus 1 (FHV-1) and feline calicivirus (FCV) (3–5). It is suggested that these primary viral pathogens, particularly FHV-1 (6), damage the epithelial mucosal barrier, making affected cats more susceptible to secondary bacterial infections involving *Mycoplasma (M.)* species, *Pasteurella* species, and *Bordetella bronchiseptica* (7), which may contribute to the development or perpetuation of chronic nasal disease. In acute URTD, empirical antimicrobial therapy is indicated in febrile, lethargic, or anorexic cats, with doxycycline recommended as the first-line agent for 7–10 days (3), due to its efficacy against the aforementioned bacteria, including *Mycoplasma felis* and *B. bronchiseptica* (8). However, its use may be limited in anorectic cats, as administration with food and/or water is recommended to minimize the risk of esophageal injury or stricture (9, 10).

The most common causes of chronic nasal discharge in cats are nasal neoplasia and chronic rhinosinusitis (CRS), accounting for 38 and 35% of cases, respectively (7, 11–13). Diagnosis relies on a multimodal diagnostic approach including computed tomography (CT) and rhinoscopy. CRS is diagnosed based on histopathologic evidence of rhinitis after exclusion of other underlying conditions (2, 11), such as foreign bodies, fungal infection, oronasal or dental disease, and neoplasia (14). The exact etiology of CRS remains unclear (15). As previously mentioned and as suggested in acute URTD, prior viral infections—such as FHV-1—in affected cats are considered a possible triggering factor, although definitive evidence is lacking (6). Additionally, although a variety of bacteria have been detected in affected cats, the primary pathogenetic role of bacterial pathogens remains controversial (11, 12, 16). Potentially pathogenic (11) bacteria in CRS cats include *Pasteurella multocida*, *Escherichia coli*, and *Pseudomonas* species (6), with multidrug-resistant strains reported in the latter two (17). However, antimicrobial treatment in cats with CRS, including reserve antibiotics targeting multidrug-resistant bacteria, typically results in only temporary improvement of clinical signs (18). Recent literature emphasizes that non-responding CRS cats fail to improve even after multiple courses of antibiotics (19) and/or targeted antibiotic therapy against identified bacteria (e.g., *Pseudomonas* spp.). Therefore, based on specific clinical or imaging findings, surgical and thus more invasive treatment approaches such as trephination have been considered in individual cases (19).

As similar bacteria have been isolated in other nasal diseases and have been evaluated as secondary, some authors have already questioned the need for a culture-based bacteriological examination [cBE; formerly referred to as culture-based microbiological examination (20, 21)] of nasal swabs (14), given its limited diagnostic yield and particularly in the context of an already costly diagnostic work-up in cats with chronic nasal disease. Additionally, the results of cBE may differ from those obtained using polymerase chain reaction (PCR) techniques (19). Some researchers debate the routine use of cBE in cats with nasal discharge or recommend interpreting the results with caution (14, 18).

Treatment outcome in CRS cats can be disappointing, prognosis is guarded, and relapses are commonly reported (2, 11). To date, no standardized, evidence-based therapeutic protocol for feline CRS has been established. As *M. felis* is considered to play a particularly significant role in some cats, in case of a suspected infection, doxycycline is administered for 19 and 20 days, as well as up to 42 days (22) in contrast to the aforementioned 7–10 days in case of acute URTD (3). In contrast to dogs with idiopathic rhinitis, for which anti-inflammatory or immunosuppressive regimens with, e.g., cyclosporine are described (23), comparable data for cats with CRS are missing. Facing the use of immunomodulatory or immunosuppressive drugs (hereinafter referred to as ‘immunosuppressives’ for simplicity), testing for viral pathogens of the upper respiratory tract using PCR techniques, may be relevant since FHV-1 infections can be reactivated by stress or corticosteroid treatment (4). Additionally, *Mycoplasma* species can be tested using these PCR techniques, as higher detection rates have been reported for the PCR in contrast to standard cBE (11, 24).

Due to increasing prescription of antibiotics, rising antibiotic resistance, the known variability in cBE results depending on sampling location and/or examination method, and the associated costs (14, 18), the present study critically evaluated the diagnostic value of cBE in cats with chronic nasal diseases, particularly during the initial work-up under anesthesia. We aimed to compare the cBE results between different nasal diseases, e.g., neoplasia, fungal disease, and CRS to evaluate its clinical relevance. Secondly, we wanted to investigate its usefulness in guiding treatment decisions in cats with CRS. Recent studies have demonstrated that antibiotics, particularly in cats, can exert long-lasting effects on the gastrointestinal tract by altering the microbiome, underscoring the need for a critical evaluation of the indication for antibiotic therapy (25, 26).

We hypothesized that cBE results are neither diagnostic nor prognostic in cats with nasal diseases. Furthermore, we assumed that some cats with CRS would respond to a 21-day course of doxycycline, despite documented bacterial resistance *in vitro*, while others would show improvement under immunosuppressive therapy, even in the presence of positive cBE results.

## Materials and methods

### Study population and ethics statement

This retrospective study was based on the review of medical records from client-owned cats that were evaluated and treated at the Small Animal Clinic of the University of Veterinary Medicine Hannover, Foundation, Germany, between 2021 and 2024. As all cases were managed as part of routine veterinary care and no experimental procedures or interventions were performed, ethical approval was not required according to institutional and national guidelines. All owners had provided informed consent for the anonymized use of clinical data for research and publication purposes.

**Figure 1:**
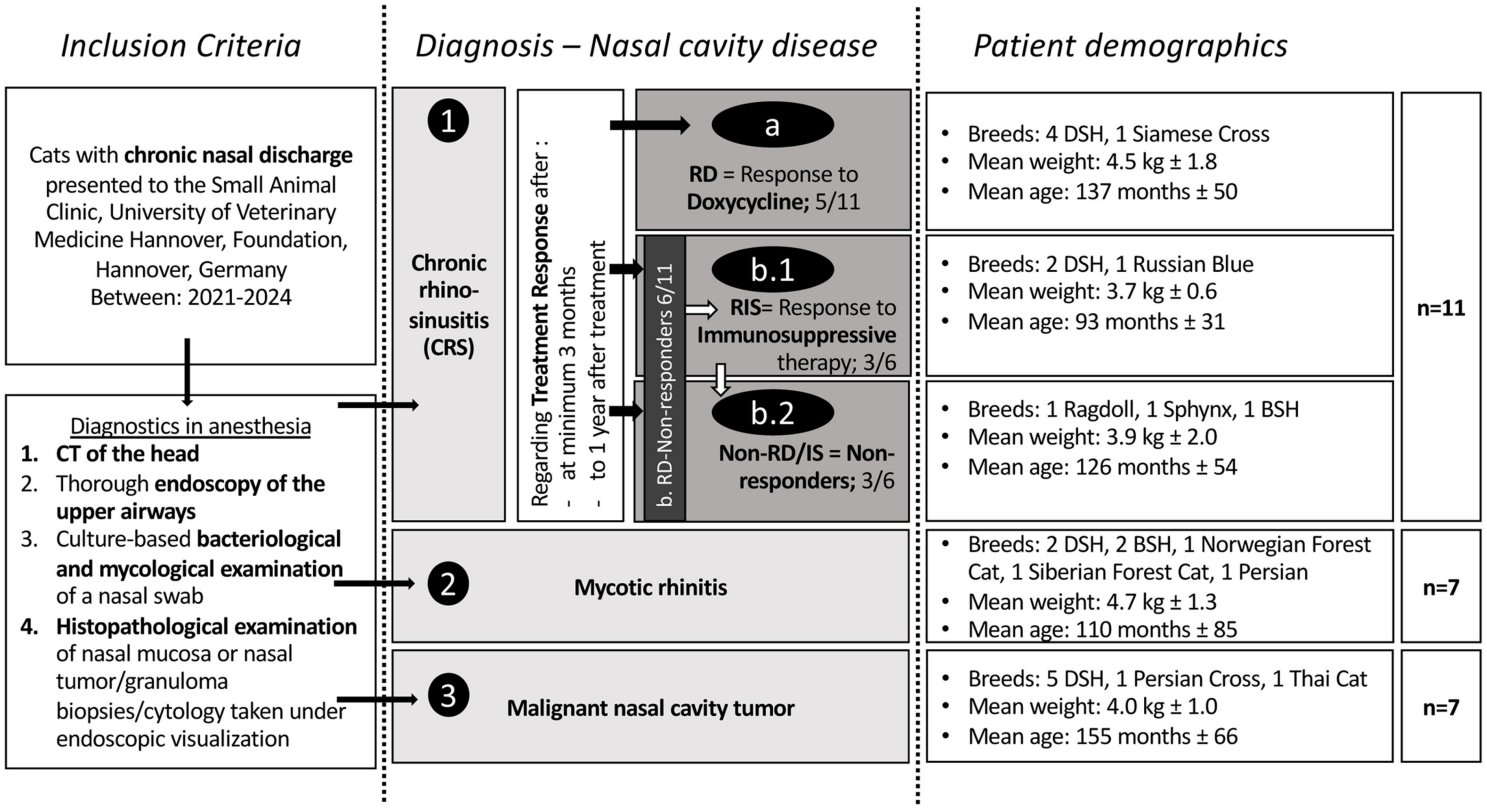
Illustration of the study design and included feline patients. Inclusion criteria were the clinical sign of chronic nasal discharge and different performed diagnostic steps. According to the diagnostic results, cats were grouped as illustrated. The groups of nasal cavity pathologies are represented in light gray: 1. Chronic rhinosinusitis (CRS; combined group with subgroups according to different treatment responses), 2. Mycotic rhinitis, and 3. Nasal neoplasia. Within the CRS group, subgroups were formed based on treatment response: a. responders to doxycycline (RD), b. non-responders to doxycycline (Non-RD) but b1. to immunosuppressive drugs (RIS; after no clinical response to doxycycline therapy) or b2. non-responder to doxycycline and to immunosuppressive therapy (Non-RD/IS). These subgroups were created to evaluate differences in culture-based findings leading to prognostic factors regarding treatment of CRS cats in veterinary practice. Additionally, basic patient demographics are depicted in the figure. The affected breeds, the weight (*p =* 0.46), and age (*p =* 0.55) were not significantly different between the groups. DSH, Domestic Shorthair; BSH, British Shorthair.

Cats were eligible for inclusion if they presented with chronic nasal discharge lasting more than two weeks (Figure 1) and if a diagnosis of nasal pathology had been established at the initial visit based on computed tomography (CT) of the head and a comprehensive endoscopic examination of the upper airways, including both anterograde and retrograde approaches. Inclusion also required available results from culture-based mycological examination, culture-based bacteriological examination (cBE) of nasal mucosa swabs, and cytological or histopathological analysis of endoscopically visible lesions, such as tumors, granulomas, or nasal mucosa biopsies.

At first presentation, nasal discharge was classified based on the retrospective clinical history provided by the owners. Due to the variability in owner descriptions and the changing nature of nasal discharge over time, three categories were defined for comparison: serous to mucopurulent discharge, mucopurulent to purulent discharge, and epistaxis.

All cats initially underwent endoscopic interventional treatment and debridement via the nasal passages—for example, in cases of chronic rhinosinusitis (CRS), nasal tumors, or fungal granulomas—performed at the discretion of the treating ENT specialist (SR). However, the details of these procedures are not focus of this manuscript.

### Follow-up of CRS cats

The response to intervention and treatment was evaluated. Follow-up imaging via CT and endoscopy under anesthesia was offered to the owners. If owners declined, follow-up was conducted through phone calls and email correspondence by two of the authors (CE and SR)—due to the distance to the referral clinic. Appropriate and comprehensive questions based on published questionnaires from dogs (27) were used, including general well-being, nasal discharge, sneezing, reverse sneezing, coughing, as well as increased licking frequency, or other abnormalities. By repeatedly following up with owners over an extended period after the initial examination and treatment, the cats were grouped according to the owners’ responses to these questions as responder (no clinical signs) or non-responder (still any kind of clinical signs). Given the inherently subjective nature of owner-reported clinical signs, comparable to evaluations in feline pain and behavior (28–30), a binary grading system (‘clinical signs: yes or no’; no subgrading of severity of clinical signs) was used to minimize bias and reduce subjectivity effectively.

### Grouping of included cats

The grouping was based on the results of the initial diagnostic work-up, as described above, and on the follow-up findings in cats with CRS:

1.  CRS: Diagnosis of exclusion (including negative mycological examination of a nasal swab), with subgroups defined according to the treatment response, as repeatedly reported by the owners, following endoscopic interventional nasal cleaning (flushing with 0.9% NaCl). Outcome of different treatments was evaluated compared to pre-endoscopic clinical signs. This was necessary because all cats initially showed mild improvement after endoscopy, but ongoing clinical signs. Endoscopic guided nasal flushing/removal of mucus, manipulation and minor bleeding during the rhinoscopy procedure may have influenced these findings. Since mild nasal signs generally persisted until specific therapy (doxycycline) was started, the outcome of different treatments was evaluated compared to pre-endoscopic clinical signs.

  a.  Responders to doxycycline therapy (RD): In the long-term (evaluated after end of treatment course in contrast to pre-rhinoscopic clinical presentation): no clinical signs were observed following treatment with doxycycline (10 mg/kg q24h or 5 mg/kg q12h for 21 days PO)—complete resolution of clinical signs. Follow up per phone was performed at least 1 year after diagnostic work up or treatment and the absence of clinical signs has been confirmed.
  b.  Non-responders to doxycycline therapy (Non-RD).

    b.1. Responders to immunosuppressive therapy (RIS): No clinical improvement was observed following completion of doxycycline therapy; however, complete resolution of clinical signs was achieved following the consecutive daily administration of anti-inflammatory and/or immunosuppressive medication, such as corticosteroids (1 mg/kg q24h PO; with transition to once-daily inhaled corticosteroids (fluticasone) if effective) or cyclosporine (5 mg/kg q24h PO). Cyclosporine treatment was initiated after evaluating the response to corticosteroids. Clinical signs recurred after discontinuation of the anti-inflammatory and/or immunosuppressive treatment but resolved again once the medication was restarted.
    b.2. Non-responders to doxycycline therapy and immunosuppressive therapy (Non-RD/IS): No improvement (as well as no worsening) in clinical signs was observed following completion of either doxycycline therapy or a course of anti-inflammatory and/or immunosuppressive treatment with corticosteroids and cyclosporine.

2.  Mycotic rhinitis: In accordance with recommendations for the diagnosis of mycotic disease in dogs (31), the diagnosis of feline mycotic rhinitis was based on at least two positive diagnostic tests, such as a positive mycological culture, a positive histopathological examination, or visualization of a mycotic granuloma during endoscopy. If the fungal species could not be reliably identified via mycological culture or histopathology, PCR was performed on biopsy specimens.
3.  Malignant nasal neoplasia: A nasal or nasopharyngeal mass was detected, and malignant neoplasia was confirmed through histopathological analysis of biopsies obtained under endoscopic visualization, as well as by immunohistochemistry in case of lymphoma.

### Pretreatment

Pretreatment conducted by referring veterinarians was evaluated with regard to the prescribed antibiotics, including the number and active ingredients. In cats with CRS, bacteria detected during the endoscopic examinations (performed under general anesthesia and sterile conditions with endoscopic guidance) were compared to results from previous cBE, if available. Earlier samples had been collected either from nasal discharge in awake animals or during prior endoscopy. The sensitivity of the detected bacterium against the antibiotics in the individual history of the cat as well as against the doxycycline was evaluated (Table 1).

**Table 1:** Overview of bacteria and antibiotics previously administered to cats with CRS that were either unresponsive to doxycycline (Non-RD) but responded to immunosuppressive therapy (RIS), or that showed no response to any treatment (Non-RD/IS).

Number of the cat in the present study	Bacterium detected by the regular veterinarian before presentation (± PCR result, if available)	Antibiotic(s) received as pretreatment	Bacteria detected in our cBE in the present study (± positive PCR result)	Antibiogram results in the present study	Group	Clinically responding to
5	*Pseudomonas aeruginosa* (susceptible to **gentamicin** and marbofloxacin)	(1) **gentamicin** (2) marbofloxacin	*Pseudomonas aeruginosa*	(1) **gentamicin: susceptible** (2) enrofloxacin: intermediate (3) doxycycline: resistant	Responder to immunosuppressives – after doxycycline failure	oral prednisolone, successful switch to inhaled fluticasone possible
6	n.a.	amoxicillin-clavulanic acid	*Pseudomonas aeruginosa*	(1) only susceptible to gentamicin (2) resistant to doxycycline		responding to either oral prednisolone, inhaled steroids or oral cyclosporine
7	n.a.	**amoxicillin-clavulanic acid**	(1) *Staphylococcus felis* (2) *Bordetella bronchiseptica* PCR: *Mycoplasma felis* positive	Ad: (1) Susceptible to all tested antibiotics including **amoxicillin-clavulanic acid** Ad: (2) Susceptible to **amoxicillin-clavulanic acid** and doxycycline		only responding to oral cyclosporine
8	*Pasteurella multocida* (detected twice prior to presentation; no antibiogram available) *Mycoplasma felis* tested repeatedly positive by PCR	(1) **amoxicillin-clavulanic acid** (2) **doxycycline** (3) **cefovecin**	*Pasteurella multocida* PCR: FHV-1 positive	Susceptible to: (1) **amoxicillin-clavulanic acid** (2) ampicillin (3) **doxycycline** (4) enrofloxacin	Non-responder to doxycycline and to immunosuppressive therapy	
9	n.a.	(1) **doxycycline** (2) **enrofloxacin** (3) **amoxicillin-clavulanic acid**	(1) *Streptococcus equi ssp. zooepidemicus* (2) *Pasteurella multocida*	(1) *Streptococcus equi ssp. zooepidemicus* susceptible to **doxycycline**; *Pasteurella multocida* intermediate to **doxycycline**, (2) Both susceptible to **enrofloxacin**, (3) Both susceptible to **amoxicillin-clavulanic acid**		
19	Negative	(1) amoxicillin-clavulanic acid (2) cefovecin (3) **enrofloxacin** (4) doxycycline (5) azithromycin	*Pseudomonas aeruginosa* PCR: FHV-1 positive	**enrofloxacin**		

In 2/3 cats with a previous cBE, the same bacteria were repeatedly detected (yellow fields). Except for one cat (orange fields), the identified bacteria were generally sensitive to the antibiotics previously administered. Nevertheless, cats were referred to our clinic due to lack of clinical improvement following antibiotic therapy. Blue: doxycycline, bold black or blue: antibiotic which the cat received prior to presentation; n.a., not available as not performed.

### Mandatory diagnostic procedures for inclusion in the present study: CT, endoscopy, nasal swab for cBE, and histopathological/cytological examination of nasal mucosa

CT was performed using the Philips IQon Spectral CT SDCT (Philips Health Care Germany) with the following parameters: 120 kV, 1 mm slice thickness, tilt 0°, and 620 ms. Mass lesions or lytic lesions, as well as dental alterations, were noted. Cats with CRS were evaluated for sinusitis by assessing soft tissue isodense filling of the sinus frontalis, recessus maxillaris, or sinus sphenoidalis. Additionally, the degree of turbinate destruction was assessed and classified into three easily distinguishable categories: mild (slight destruction, difficult to discern), moderate (clearly visible destruction), and severe (complete absence of nasal turbinates within the nasal cavity, with possible presence of the ethmoidal concha).

Endoscopic examination of the upper airways was conducted following standardized protocol and without irrigation, using semiflexible NanoScope™, Console Vet, and handpiece Kit (Arthrex, Munich, Germany) or the HOPKINS® Optics, 0° (1.9 mm) and 120° (4 mm), of Karl Storz (Tuttlingen, Germany). Anterograde 0° endoscopy was used to examine the upper respiratory tract in detail. After sterile collection of nasal mucosal swabs for cBE and mycological examination, nasal discharge was removed with a suction pipe, and nasal cavities were explored in more depth. All instruments were inserted parallel to the endoscope, not through a guiding shaft. For biopsy collection, forceps were inserted parallel to the endoscope and directed to the area of interest under endoscopic visualization. If fluid- or tissue-isodense filling in one of the paranasal sinuses was suspected, an endoscopic interventional opening of the sinus was performed. The content was then evaluated, and material or secretion was cleaned and removed.

During the endoscopic examination, images were taken at specific nasal landmarks including (a) the nasal entrance, (b) within the nasal cavity, (c) the nasal exit, and (d) the nasopharynx, to enable retrospective analysis. These images were subsequently evaluated in CRS cats by the authors CE and SR. In CRS cats, increased redness (yes/no) and turbinate destruction were assessed. Turbinate destruction was graded as mild (only minimal destruction), moderate (clearly visible with markedly widened air spaces), or severe (marked loss of larger turbinate structures, particularly of the ventral turbinates in the rostral and mid-nasal cavity), and the findings were compared with the CT-based assessment of turbinate destruction. The type of nasal secretion was described by owners of CRS cats as ranging from serous-mucopurulent to purulent. As the color of the discharge in the different cats appeared nearly comparable and changed sometimes from the nasal entrance to the nasal exit, ranging from more serous to purulent, and as endoscopic color grading can be affected by lighting in the endoscopy room and white balance, no further color comparisons were made.

The amount of nasal discharge was subjectively graded on a binary scale as either low (limited to the nasal entrance and/or nasal cavity) or moderate-to-high (+ extending into the nasopharynx). In videos where nasal secretions were suctioned from the nasal cavity, they were qualitatively assessed and simply categorized as either low-viscosity (water-like) or increased-viscosity secretions.

The mucosal swabs were stored in a standard transport medium and analyzed at the Institute of Microbiology at the University of Veterinary Medicine Hannover. Bacterial culture results were evaluated both on a semi-quantitative basis (categorized as low, moderate, or high bacterial growth, *data not shown*) and on a binary level (positive cBE: yes/no; as depicted in the figures of the present study) to allow for overall comparison across groups and assessment of the presence of specific bacterial species in each disease group. If fungal infections were detected by culture or histopathology, but the species could not be identified, further PCR testing was retrospectively performed at the National Consiliary Laboratory for Cryptococcosis and Rare Systemic Mycoses (FG16 Diagnostic Mycology), Robert Koch Institute, Berlin, Germany. Histopathological examination was performed on biopsies of nasal mucosa or tumor or granuloma tissue. In cats with rhinitis, nasal mucosal biopsies were collected from the ventral concha, either unilaterally or bilaterally. If there was severe destruction of the turbinates, cytological evaluation of a nasal swab was a possible alternative to obtain information about the type of inflammation. In the case of malignant lymphoma, immunohistochemistry was performed to determine the cell type (B-cell or T-cell lymphoma).

### Possible additional diagnostic tests

Additional diagnostic tests, although not mandatory for inclusion, included the following: complete blood count and biochemical analysis performed using the in-house Advia 2,120 Hematology System (Siemens Healthineers, Germany) and the Cobas C311 Autoanalyzer (Roche Diagnostics, Germany); CT scan of the thorax; PCR testing for feline upper respiratory disease complex pathogens using combined conjunctival, nasal, and pharyngeal mucosal swabs, including FHV-1, FCV, *Mycoplasma* species, *Bordetella bronchiseptica*, and *Chlamydia felis* (IDEXX, Ludwigsburg, Germany); tests for feline leukemia virus antigen (FeLV) and antibodies against feline immunodeficiency virus (FIV), using the SNAP FIV/FeLV Test (IDEXX, Ludwigsburg, Germany). Serum tests for anti-*Aspergillus* antibodies and *Cryptococcus* antigen were conducted at Laboklin GmbH & Co. KG (Bad Kissingen, Germany).

### Statistical analysis

Statistical analyses were performed using GraphPad Prism (v10 GraphPad Software, La Jolla, CA, United States). Data were tested for normality using the D’Agostino & Pearson Normality Test and the Shapiro–Wilk Normality Test. Normally distributed data were specified with mean ± standard deviation (SD), while non-parametric data were specified with median and interquartile range (IQR). The IQR represents the range between the 25th and 75th percentiles. Comparison among parametric data was made after testing for equality of variance by Brown-Forsythe test and Bartlett’s test, by One-way ANOVA or among non-parametric data by Kruskal-Wallis Test, with either the Tukey’s multiple comparisons test if parametric data or Dunn’s multiple comparisons test if non-parametric data. For pairwise comparison of two parametric data, the unpaired t-test was used and in case of non-parametric data the Mann–Whitney test. A *p*-value < 0.05 was considered significant.

## Results

### Study population

Twenty-five cats were included in the study. Of these, 11 were diagnosed with CRS, seven with mycotic rhinitis, and seven with neoplasia (Figure 1). Patient demographics are shown in Figure 1. The median duration of clinical signs was 4 months (IQR: 1–13.3 months), with no significant differences observed between the subgroups (*p* = 0.11). Serous to mucopurulent discharge was observed in 5 cats (CRS *n* = 3, mycotic rhinitis *n* = 1 and neoplasia *n* = 1), epistaxis in 5 cats (mycotic *n* = 2, neoplasia *n* = 3) and mucopurulent to purulent in the remaining 15 cats (CRS *n* = 8, mycotic rhinitis *n* = 4, neoplasia *n* = 3).

In the group of cats with a mycotic infection, *Aspergillus* species were detected in 6/7 cats (species-level identification via PCR in 3/6 cases) and *Cryptococcus neoformans* in 1/7 cats (32). In all seven cats, fungal structures were identified on histopathological examination. Culture-based mycological examination for detecting fungal disease was positive in only 4/7 cats (57%). Serum anti-*Aspergillus* antibodies were analyzed in two cats with aspergillosis—with two positive results, while *Cryptococcus* antigen testing was performed in four cats, with one positive result in the cat with *Cryptococcosis*. In all cats within this group, antifungal treatment without the use of antibiotics resulted in a positive therapeutic response (*data not shown*).

In the group of cats diagnosed with neoplasia, one cat (1/7) was diagnosed with adenocarcinoma and 6/7 cats with malignant lymphoma, including B-cell lymphoma in four cases. All cats in this group received neoplasia-specific treatment without antibiotics, including chemotherapy, radiation therapy, or endoscopic intervention, resulting in either a partial or complete therapeutic response (*data not shown*).

CRS cats were categorized based on treatment outcomes, as described above, depending on follow-up data.

### Follow-up data of CRS cats

Follow-up was performed in 2/11 cats (both CRS: one RD and one RIS) using CT and endoscopy of the upper airways, while in one cat (1/11; Non-RD/IS) re-examination was conducted only by endoscopy due to financial constraints. Diagnostic findings were consistent with the owners’ assessment of clinical signs.

In eight out of 11 cats, owners did not consent to further examinations under general anesthesia, as described in the Materials and Methods section. Follow-up information was obtained through close communication with the owners via multiple telephone calls and/or emails, particularly for cats in the RIS and Non-RD/IS groups. These cats underwent stepwise treatment after doxycycline therapy, starting with oral corticosteroids (with inhaled cortisone added or substituted in case of a positive response), followed by cyclosporine therapy, either as a corticosteroid-sparing alternative in responsive cases to minimize long-term side effects, or as a next step when no clinical improvement with corticosteroids was observed. Due to this stepwise treatment approach, continuous contact with the owners was maintained to provide detailed information about specific medications and the way of their administration. Additionally, blood test results performed by the cats’ regular veterinarians after initiation of medication (corticosteroids or cyclosporine) were shared and discussed with us, further facilitating long-term follow-up.

Follow-up lasted at least 1 year after the initial diagnostic work-up or treatment.

Because of the binary grading system, only cats that showed a complete response to one specific medication were included in the respective subgroups. Cats with ongoing clinical signs or relapses received further treatment and/or changes in medication were categorized, e.g., in the group non-responders. Importantly, there were no conflicting assessments (e.g., improvement at 3 months followed by relapse at 9 months). Notably, not all cats in the RS-IS group that responded to cyclosporine had previously responded to corticosteroids.

### Grouping of cats

1.  CRS (11/25).

  a.  Responders to doxycycline therapy (RD; 5/11–45%): Five cats were assigned to the CRS doxycycline responders group, as they showed no respiratory signs at all after treatment, which was confirmed even after at minimum 1 year of treatment.
  b.  Non-responders to doxycycline therapy (Non-RD; 6/11–55%), with subsequent.

    b.1.  Responders to immunosuppressive therapy (RIS; 3/6–50% of Non-RD): Three cats that did not respond to various antibiotics during pretreatment, nor to doxycycline treatment, showed a positive response to long-term treatment with anti-inflammatory drugs or immunosuppressive therapy. Two of these cats responded to cyclosporine (5 mg/kg q24 hours), but experienced relapses after discontinuation. In one cat, treatment was discontinued due to financial constraints related to the high cost of the medication cyclosporine and difficulties with oral administration; in the other, treatment was stopped solely for financial reasons. In both cases, clinical signs resolved after cyclosporine was reintroduced with 5 mg/kg q24 hours. For the former cat, cyclosporine was administered as a liquid formulation (Sporimune® 50 mg/mL oral solution, Dechra Veterinary Products GmbH, Aulendorf, Germany) for exact dose calculation, encapsulated in empty capsules as described elsewhere (33). In the latter case, this method proved impractical, so the cat was switched to a higher-dose regimen (Atopica® 25 mg; 6 mg/kg body weight, Elanco, Bad Homburg, Germany) administered on a Monday–Wednesday–Friday (MWF) schedule after several weeks of daily administration. Under this regimen, the cat remained asymptomatic. However, further dose reduction was not possible without recurrence of clinical signs. Of these two cats, one had responded initially to oral corticosteroids (prednisolone 1 mg/kg q24 hours) after a treatment trial with doxycycline, as well as to inhaled fluticasone, but had skin reactions surrounding the nose when using the inhaler (suspected non-infectious alopecia, resolving after discontinuation). Therefore, cyclosporine therapy was initiated. The other one did not respond to steroids before administering cyclosporine. The third cat responded well to oral prednisolone (prednisolone 1 mg/kg q24 hours) as well as inhaled fluticasone alone, which is why treatment with cyclosporine was not initiated.
    b.2.  Non-responders (Non-RD/IS; 3/6–50% of Non-RD): Three cats that did not respond to various antibiotics during pretreatment were classified as non-responders to both a three-week course of doxycycline and a four-week trial of each immunosuppressive treatment. For corticosteroids, this included two weeks at 1 mg/kg body weight once daily, followed by a two-week tapering phase. In cases of slight clinical improvement, the evaluation period was extended; however, a sustained response was not confirmed. Some owners concurrently used nebulized air and mucolytic therapy, which may have contributed to temporary improvements. Nonetheless, these cats did not achieve the complete response observed in cats of groups a or b.1. Importantly, none showed worsening of clinical signs under either treatment regimen.

2.  Mycotic rhinitis (7/25).
3.  Malignant nasal neoplasia (7/25).

### Pretreatment

Of the 25 enrolled cats, 21 (84%) had received pretreatment with one or more oral antibiotics (RD CRS median number antibiotics 1 [IQR: 0–1], RIS CRS 1 [IQR: 1–4], Non-RD/RIS CRS 3 [IQR: 3–3], mycotic 1 [IQR: 1–2], neoplasia 2 [IQR: 1–2]). Four cats (16%) had not received any antimicrobial therapy prior to further diagnostics, including two CRS cats responding to doxycycline (RD), one cat with nasal neoplasia, and one with a mycotic infection. The number of antibiotics was statistically significantly different between groups (*p* = 0.05): between RD CRS cats and the Non-RD/IS CRS cats (*p =* 0.03). In general, a median of two different antibiotics were administered per cat (IQR: 1–2). The most frequently used antibiotics were doxycycline and amoxicillin-clavulanic acid, each prescribed to nine cats, followed by amoxicillin (*n* = 4), enrofloxacin (*n* = 4), and cefovecin (*n* = 4). Other antibiotics included marbofloxacin (*n* = 3), gentamicin (*n* = 1), metronidazole (*n* = 1), clindamycin (*n* = 1), cephalexin (*n* = 1), and unspecified antibiotics (*n* = 2). While one cat with a nasal neoplasia had received a dexamethasone injection three days prior to diagnostics, and one cat with RD-CRS was on long-term cyclosporine therapy (5 mg/kg administered every other day) for an unspecified allergic skin condition, none of the cats was receiving antibiotics at the time of endoscopy, nor had they received any antibiotic treatment for at least 1 week prior to the procedure.

For example, Table 1 presents the data of the Non-RD CRS cats with subgroups RIS and Non-RD/IS cats. Illustrated are results of cBE performed by the referring veterinarians, if available, the previously prescribed oral antibiotics, the results of our own cBE with susceptibility profiles. All cats were pretreated with antibiotics, and except for one cat from the RIS subgroup, all cats had previously received antibiotics to which the bacteria identified in our analysis (in two cases repeatedly) were likely susceptible, yet without showing any clinical improvement. All cats in the Non-RD subgroup had previously received doxycycline without showing any clinical improvement; however, the duration of treatment had been shorter than in the present study (thereby maybe not effective against *Mycoplasma* species), which is why treatment with doxycycline over 21 days was repeated, without clinical improvement.

Two Non-RD/IS cats that tested positive for FHV-1 received oral famciclovir (in unknown dosage), one prior to presentation (as well as feline recombinant omega interferon, L-lysine hydrochloride), and the other later in the course of the disease following diagnostics at our clinic, with no significant impact on treatment outcome.

### CT findings in CRS cats

Of the 11 cats enrolled in the CRS group, only 10/11 (90.9%) showed changes compatible with rhinitis on CT including fluid accumulation and/or turbinate destruction, although bilateral rhinitis was confirmed by histopathology in all cats (11/11; 100%). Nasal turbinate destruction was observed in 9/11 cats, ranging in severity: mild in five cats (RD 3/5, RIS 2/3), moderate in 1/3 of the RIS group, and severe in 3/3 of the Non-RD/IS group. The sample size of cats without turbinate destruction was insufficient to allow for meaningful statistical analysis of potential associations with specific bacterial species.

Paranasal sinus involvement was observed in 9/11 cats (81.8%), characterized by fluid- to soft-tissue-attenuating, non-enhancing material. Two cats of the RD group showed no evidence of sinusitis (18.2%). Most affected cats (7/11; 77.8%) exhibited involvement of multiple paranasal sinuses. The maxillary recess was the most often affected sinus, observed in 9/11 cats (bilateral involvement in four cats, unilateral involvement in five cats). The frontal sinus was affected in 5/11 cats (46%; bilateral involvement in two cats, unilateral involvement in three cats), and the sphenoid sinus in 4/11 cats (36%; bilateral involvement in two cats, unilateral involvement in two cats).

### Endoscopic findings in CRS cats

**Figure 2:**
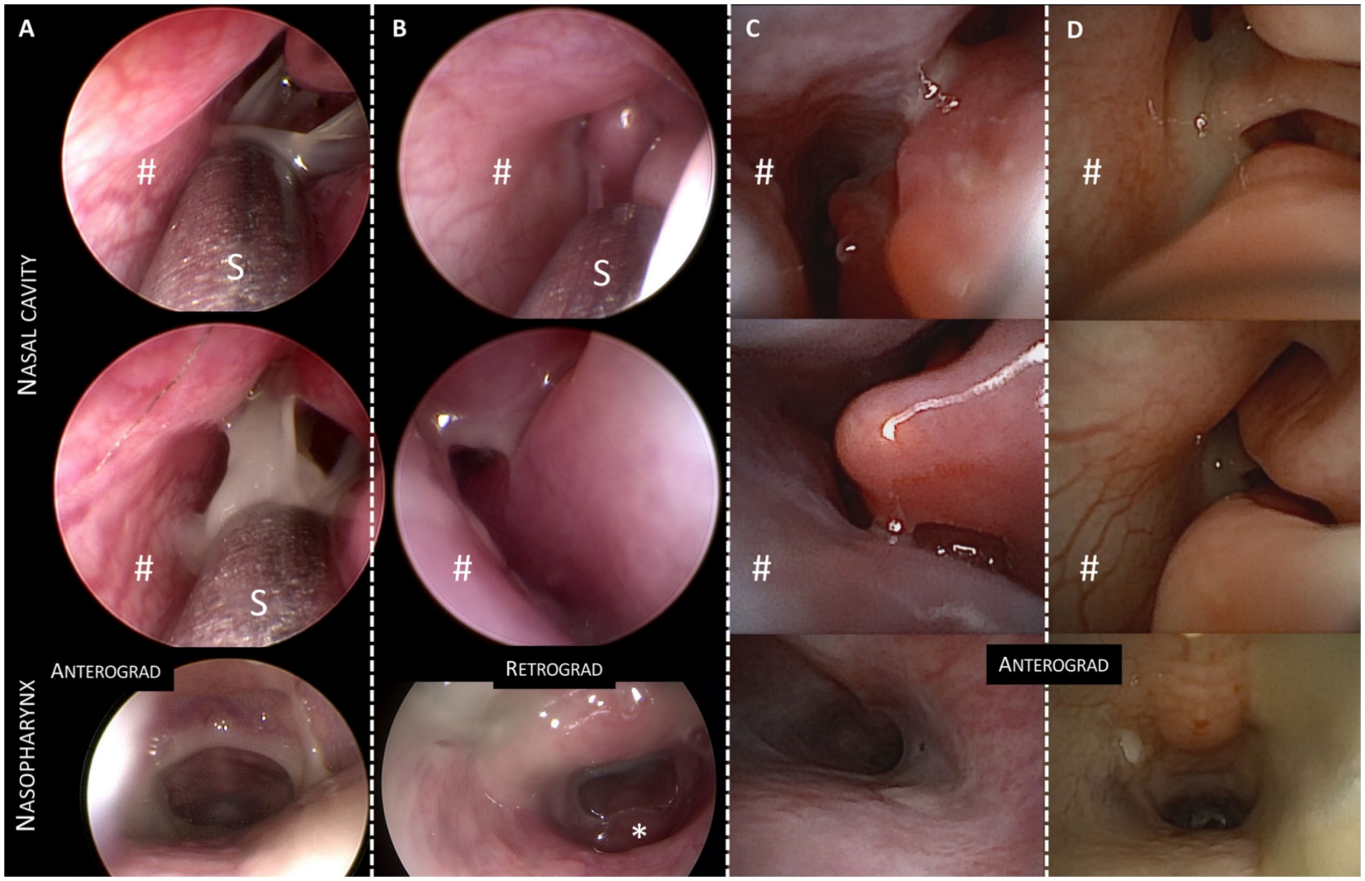
Endoscopic images of four cats with chronic rhinosinusitis (CRS). Each column **(A–D)** presents three images of one cat (two of the nasal cavity and a third from the nasopharynx showing nasal/nasopharyngeal secretion). Cats **(A–C)** belong to the CRS subgroup that responded to doxycycline, demonstrating varying amounts of serous and yellow secretion. In contrast, images in column **D** represent a cat with CRS that responded to immunosuppression. These images do not show significant differences in the endoscopic appearance of the amount or color of nasal/nasopharyngeal secretion compared to the doxycycline responders. #nasal septum; *tonsilla pharyngea at the skull base in the retrograde image; S = metal suction pipe. Two different endoscopes were used in the cats: **A,B**: HOPKINS® Optics, Karl Storz (Tuttlingen, Germany); **C,D**: NanoScope™, Console Vet and handpiece Kit (Arthrex, Munich, Germany).

In all cats, increased viscous secretions and varying degrees of mucosal redness were detected, as well as turbinate destruction. Importantly, no significant differences in amount of fluid, the degree of yellow color, or viscosity were detected between cats of different subgroups of CRS regarding therapeutic outcome (Figure 2). In contrast, severe forms of turbinate destruction were only seen in Non-RD/IS.

### Detected bacteria—culture-based bacteriological examination (cBE) and PCR

**Figure 3:**
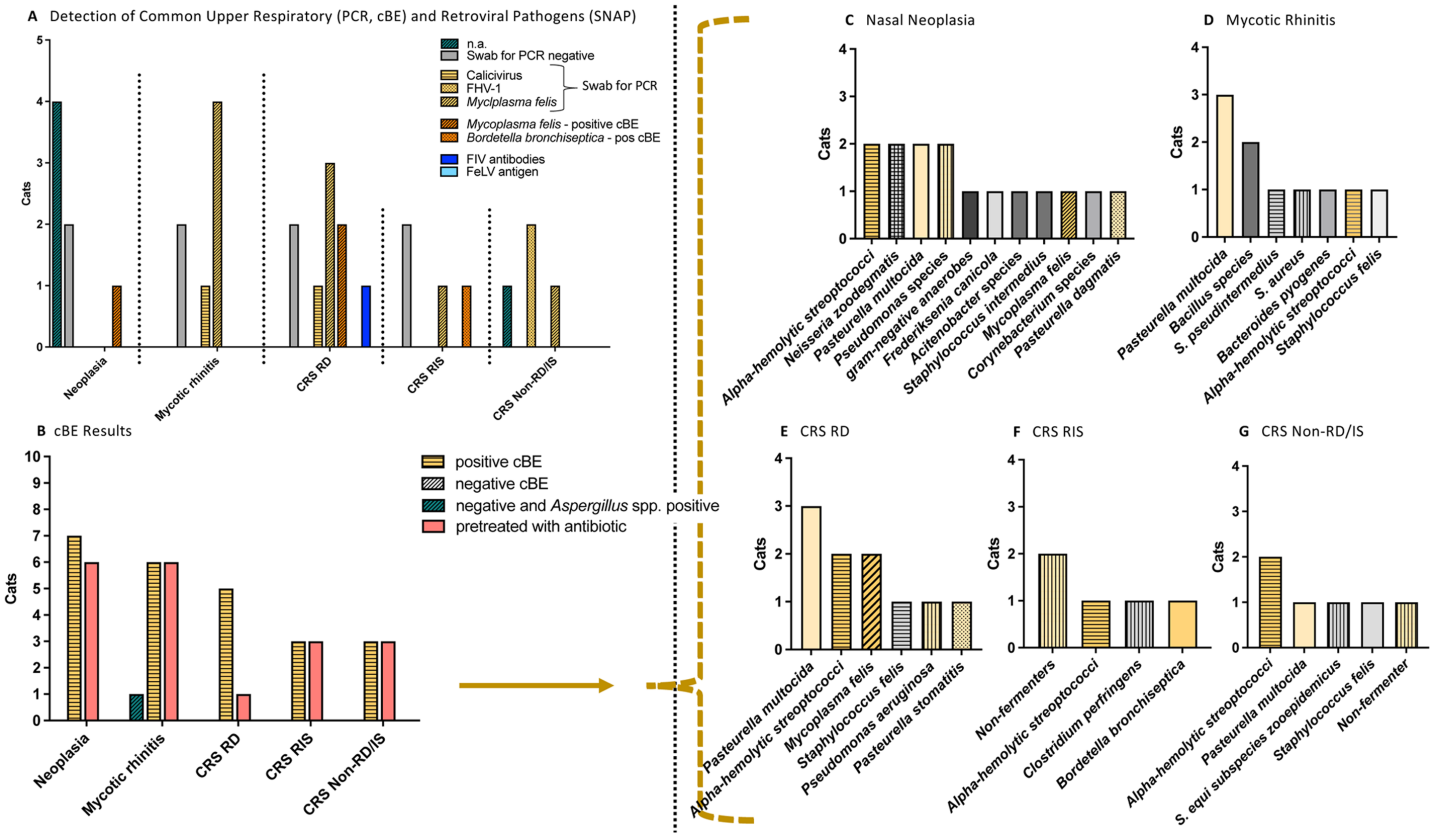
Results of PCR results, FIV/FeLV tests and culture-based bacteriological examination (cBE) in cats with nasal diseases. Nasal cavity diseases were nasal neoplasia (*n* = 7), mycotic rhinitis (*n* = 7) and chronic rhinosinusitis (CRS; *n* = 11) [subgroups: CRS RD = Responded to doxycycline: Clinical response/improvement to doxycycline therapy (*n* = 5), Non-responders: with CRS RIS = Responded to immunosuppression (*n* = 3), or CRS Non-RD/IS = Non-responder: Pretreated with antibiotics; no response to doxycycline or to immunosuppression (*n* = 3)]. **(A)** Illustration of PCR results and FIV/FeLV tests. Only one cat (CRS RD group) was tested positive for FIV antibodies. **(B)** The cBE was (in both ways of evaluation: binary or semiquantitative) positive in all cats except one, in which cBE was negative and fungal organisms were detected. Except for 4/25 cats (two cats of the RD group, one cat with neoplasia, and one cat with mycotic rhinitis), all cats had previously received antimicrobial treatment. No antimicrobial was given for at least one week before evaluation. **(C–G)** Overview of the most bacterial organisms identified in the cBE. n.a., not applicable; FHV-1, Feline Herpesvirus type 1; PCR, polymerase chain reaction; FIV, Feline Immunodeficiency Virus; FeLV, Feline Leukemia Virus.

**Figure 4:**
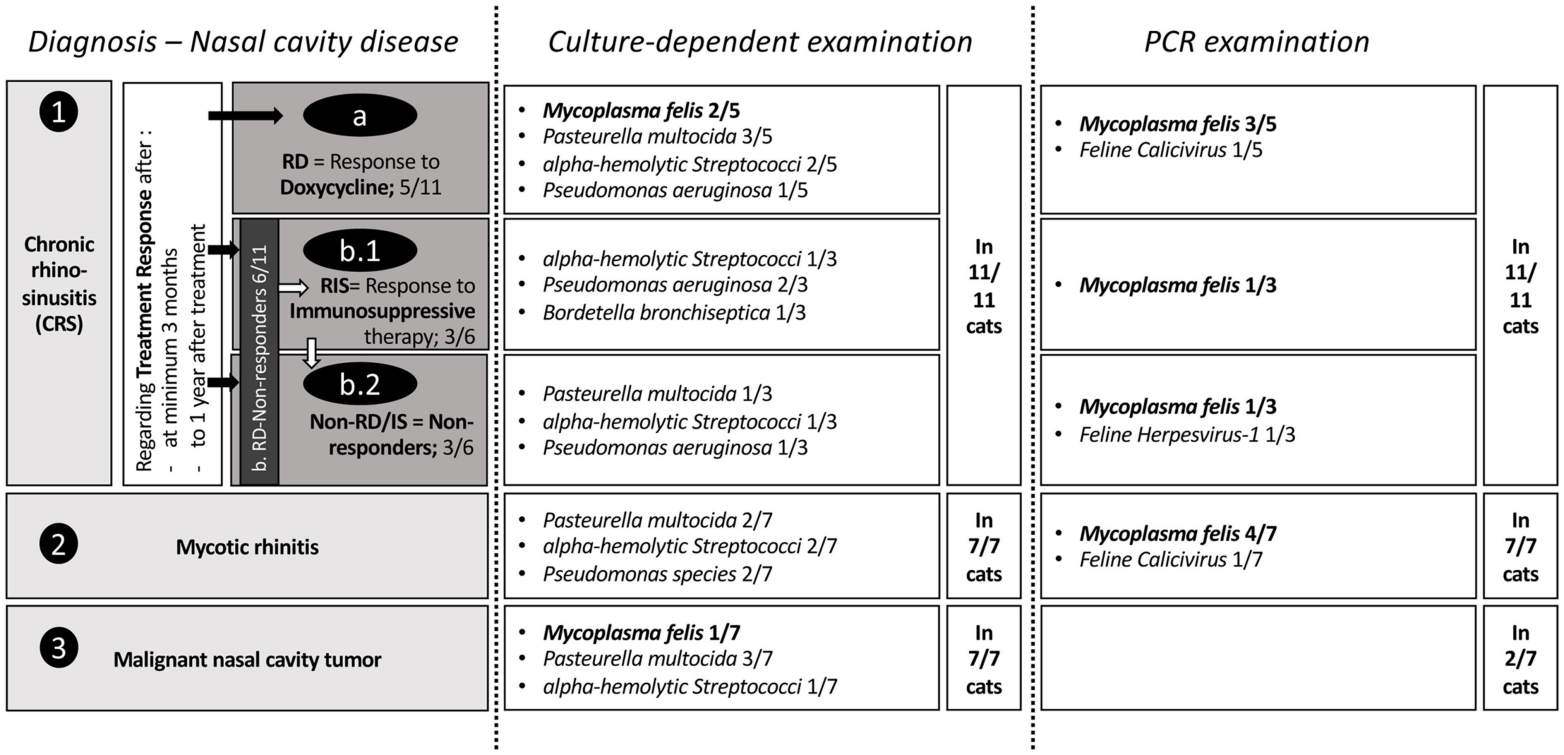
Illustration and comparison of the positive results of bacteriological examination (cBE) and polymerase chain reaction (PCR) in cats with different nasal diseases with special focus on *Mycoplasma felis*. On the right side in the second and third column, next to the result field of the detected bacteria, information is given, how many cats this examination was performed. Therefore, it is important to highlight that the PCR examination was only conducted in 2/7 cats with nasal neoplasia, with both cats being negative. No significant differences between nasal bacteria were detected with cBE in different disease groups. Consistent with findings in the literature, PCR detected a higher number of *Mycoplasma felis*-positive cats compared to cBE.

Despite antibiotic pretreatment in 21/25 cats at various time points before presentation, cBE yielded positive results in all but one cat (24/25), which tested negative for bacteria but positive for *Aspergillus* species (Figures 3, 4). Therefore, all CRS cats and cats with nasal neoplasia, as well as 6/7 cats with mycotic disease, showed a positive cBE. In a binary evaluation with positive cBE yes/no, the detected bacteria did not differ in bacterial quantity per cat across all groups and subgroups (*p* = 0.44; RD CRS median 4 [IQR: 1.5–5.5], RIS CRS 3 [IQR: 2–5], Non-RD/RIS CRS 3 [IQR: 3–4], mycotic 2 [IQR: 1–4], neoplasia 2 [IQR: 1–3]).

When evaluating the detected bacterial species, a variety of bacteria were identified, with no significant differences in species distribution among the three groups (Figures 3, 4).

*M. felis* was the most frequently detected bacterium, found in 10/25 cats using PCR and/or cBE (40%; Figures 3, 4; Table 2). This bacterium was present in cats from all three subgroups (5/11 CRS cats: 3/5 RD, 1/3 RIS, 1/3 Non-RD/IS, 4/7 mycotic rhinitis and 1/7 neoplasia). Seven of 10 cats (70%) tested positive by PCR while cBE was negative. *M. felis* was identified in 2/10 cats (20%; from the CRS RD group) by both culture and PCR methods; while in one cat with neoplasia, *M. felis* was detected via culture, but PCR screening was not performed due to financial constraints (Figure 4). A direct comparison between detection rates using cBE and PCR was not possible, as not all cats underwent both tests.

**Table 2:** Examples of previous reported bacteria detected in nasal swabs from cats with chronic rhinosinusitis (CRS; synonyms used in the cited literature include chronic rhinitis and rhinitis) by culture-based bacteriological examination (cBE) indicating comparable findings.

Author & Citation	Nasal disease in examined cats	Cultivated bacteria in cats with CRS
Johnson et al. (6)	17 Cats 58.8% Chronic rhinosinusitis	*Pasteurella multocida (30%)* *Pseudomonas aeruginosa/ Non-fermenter group (20%)* *Mycoplasma species (20%)* *Coagulase-negative Staphylococcus (30%)*
Niedenführ et al. (18)	21 Cats 81% Chronic rhinitis	*Pasteurella multocida (47%)* *Staphylococcus felis (35%)* *Staphylococcus haemolyticus (18%)* *Staphylococcus epidermidis (12%)* *Neisseria zoodegmatis (29%)* *Neisseria* spp. *(12%)*
Meepoo et al. (17)	395 Cats 36.7% Rhinitis	*Pseudomonas* spp. (32%)/ *Non-fermenter group* *Pasteurella* spp. (24%) *Staphylococcus* spp. (18%) *Escherichia coli* (8%) *Klebsiella* spp. (5%)
Present study	25 Cats 44% CRS	*Mycoplasma felis* (cBE 18%; PCR and cBE 46%) *Pasteurella multocida* (36%) *Staphylococcus* spp. (36%) *alpha-hemolytic streptococci* (46%) *Pseudomonas* spp. (36%)/ *Non-fermenter group* *Staphylococcus felis* (27%) *Bacillus* spp. (9%)

The results of the present study in this table represent results of a binary evaluation of cBE (i.e., whether the pathogen was detected or not). The use of the term rhinosinusitis is justified in the present study, since “sinusitis” in this context does not refer exclusively to involvement of the frontal sinus. Notably, nearly 80% of our CRS cats showed involvement of one or more paranasal sinuses (see CT section).

Other commonly detected bacteria were *Pasteurella multocida* in 9/25 cats (36%; in 4/11 CRS cats: 3/5 RD, 1/6 Non-RD/IS, in 3/6 cats with mycotic rhinitis and 2/7 cats with neoplasia); *alpha-hemolytic Streptococci* in 8/25 cats (32%; in 5/11 CRS cats: 2/5 RD, 1/3 RIS, 2/3 Non-RD/IS, 1/7 cats with mycotic rhinitis and 2/7 cats with neoplasia), and *Non-fermenters* (formerly classified as *Pseudomonas* species) in 7/25 cats (28%; in 4/11 CRS cats: 1/5 RD, 2/3 RIS and 1/3 Non-RD/IS, 1/7 mycotic rhinitis and 2/7 neoplasia) and *Staphylococcus species* in 9/25 cats (36%; in 5/11 CRS cats: 2/5 RD, 1/3 RIS, 1/3 Non-RD/IS, 4/6 mycotic rhinitis and 1/7 neoplasia).

*Staphylococcus* species included (note: one cat showed more than one Staphylococcus species): *Staphylococcus felis* in 4/9 cats (3/11 CRS cats: 1/5 RD, 1/3 RIS, 1/3 Non-RD/IS, 1/6 mycotic rhinitis), coagulase negative *Staphylococcus* in 2/9 cats (1/11 CRS: 1/5 RD; 1/6 mycotic rhinitis), *Staphylococcus pseudointermedius* in 1/9 (mycotic rhinitis), *Staphylococcus equi subspecies zooepidemicus* in 1/9 (CRS Non-RD/IS), *Staphylococcus aureus* in 1/9 (mycotic rhinitis) and *Staphylococcus intermedius* in 1/9 (neoplasia).

Importantly, bacteria resistant to doxycycline were detected in cats of the RD group of the CRS cats: *Serratia marcescens* and *Non-fermenters.*

### Histopathological examination of the nasal mucosa and association with specific bacterial pathogens

Histopathological examination results of nasal mucosa biopsies were available for all CRS cats except one from the Non-RD/IS group, where due to high-grade turbinate destruction, only cytological examination of nasal mucosa swabs was conducted. Lymphoplasmacytic inflammation was observed in two cats of the RD group (as well as in 1/7 cats with mycotic rhinitis). A neutrophilic, ± necrotizing type of inflammation was identified in two CRS cats: one of the RD group and one of the RIS group (as well as in two cats with mycotic rhinitis and one cat with neoplasia). Neutrophilic inflammation combined with lymphocytic, lymphoplasmacytic, or lymphohistiocytic components were detected in four CRS cats, including two of the RD group and two of the Non-RD/IS group (as well as in one mycotic rhinitis and in 4 cats with neoplastic diseases). Other types of mixed-cell inflammation were diagnosed in two cats of the RIS group and one of the Non-RD/IS group (as well as in two cats with mycotic rhinitis). The type of inflammation did not show a correlation with any specific bacterium detected (*data not shown*).

### Additional diagnostic tests

PCR testing for pathogens associated with the feline upper respiratory disease complex was performed in 19 out of 25 cats (76%; including testing for 992 *B. bronchiseptica* in 16/25; Figure 4). Screening was not conducted in 6/25 cats (24%), including one cat of the Non-RD/IS group and five cats with nasal neoplasia due to the financial burden of the owners. All cats tested for *Chlamydia felis* and *B. bronchiseptica* returned negative PCR results. Two cats tested positive for FCV (RD *n* = 1, mycotic infection *n* = 1) and two tested positive for FHV-1 (Non-RD/IS *n* = 2). As previously described, PCR testing identified *Mycoplasma* species in 9/19 cats: 3/9 in the RD group, 1/9 in the RIS group, 1/9 in the Non-RD/IS group, and 4/9 in the mycotic infection group.

FeLV and FIV testing was performed in 16/25 cats (64%). Of these, one cat (6%) tested positive for FIV. The FIV-positive cat, diagnosed with CRS, showed a favorable response to doxycycline treatment.

A complete blood count and serum biochemistry were available for all cats, except one in which biochemical analysis was not carried out. No clinically relevant abnormalities were observed in any of the cats. The albumin-to-globulin ratio (AGR, *n* = 24), eosinophil count, neutrophil-to-lymphocyte ratio (NLR, *n* = 19), and platelet-to-lymphocyte ratio (PLR, *n* = 19) did not differ significantly among the various subgroups (*p =* 0.25, *p =* 0.39, *p =* 0.83, *p =* 0.71, respectively).

## Discussion

In this retrospective study, 25 cats with chronic nasal diseases were evaluated using a comprehensive multimodal diagnostic approach including upper airway endoscopy and CT, both of which significantly enhance diagnostic accuracy and reliability (20). Bacterial species were detected by cBE in 24 of 25 cats diagnosed with either CRS, fungal infection, or neoplasia. 21/25 cats had been pretreated with different antibiotics, including reserve antibiotics. This finding should be carefully considered in the context of antimicrobial stewardship, particularly regarding the empirical use of reserve antibiotics, and the known long-lasting pathological effects of antibiotics on the feline intestinal microbiome (25, 26).

When addressing our first aim, distinguishing between different nasal diseases by comparing cBE results, we found that the detected bacterial species did not differ significantly among nasal disease groups (Figure 3; using binary or semiquantitative approaches for evaluation). As summarized in Table 2, the bacterial isolates identified in our study are comparable with those reported in the literature for cats with various nasal diseases, whether obtained using culture-based methods (17, 18) or next generation sequencing approaches (7). Our data highlight that a positive cBE result can occur alongside neoplastic, inflammatory and mycotic diseases, and that the same bacterial species can be present in the nasal cavity regardless of the underlying disease. This finding underscores that performing cBE alone—without further diagnostic work-up—in cats with chronic nasal disease based solely on the suspicion of a primary bacterial infection may lead to misdiagnosis and delay in identifying underlying neoplastic or fungal disease. Additionally, primary diseases like fungal infections or neoplasia typically require treatments other than antibiotics, as was the case in the present study.

The pathogenesis of CRS still requires clarification; consequently, the role of the detected bacteria in nasal samples remains unclear (2). Comparable to fungal or neoplastic diseases, where isolated bacteria are considered secondary to the primary disease, there are several factors that support the hypothesis that this is also the case in CRS, despite the primary cause of the disease being unknown (11). This is further supported by the present study, in which most cats had received antibiotic treatment(s) without clinical improvement, as has also been reported in current literature (19). Many other studies report transient or absent responses to antibiotic therapy in cats with CRS, even if the selected antibiotics were based on *in vitro* susceptibility testing (6). In this context, it must be considered that the administration of oral antibiotics in cats can be challenging for the owner (34). Specific consideration has to be given to *Mycoplasma* species, a bacterium in need for specific antibiotic therapy and being a relevant pathogen in URTD, particularly in post-viral or co-infection settings. In two CRS cats of the present study where *M. felis* was detected by cBE, clinical improvement was observed following doxycycline treatment, suggesting its potential role in clinical disease. As previously reported in the literature (11), *Mycoplasma* species were more frequently detected by PCR than by cBE, which raises questions about the utility of cBE for its detection, especially if PCR results are available. However, it is very important to note that *M. felis* can be found in asymptomatic cats, with reported detection rates ranging from 30 to 93% in oropharyngeal swabs (16). In the present study, these pathogens were also detected in cats with nasal diseases other than CRS, including one cat with nasal neoplasia (even in cBE), and in four out of seven cats diagnosed with mycotic disease via PCR. This latter group of cats improved clinically following treatment following the underlying fungal infection rather than for bacteria or *Mycoplasma* species. This casts doubt on the role of this pathogen in the course of disease. In contrast, in the lower respiratory tract, *Mycoplasma* species act as primary pathogen, causing inflammatory conditions in the pulmonary parenchyma and pleura (35).

Findings of the present study regarding specific pathogens: *B. bronchiseptica* is often reported to be a primary pathogen in cats but also frequently isolated as a common commensal (14). In the present study, *B. bronchiseptica* was detected in a cat with CRS by cBE. This cat did not respond to doxycycline therapy but showed a positive response to immunosuppressive therapy, thus casting doubt on the pathogen’s primary role.

*Non-fermenters* are aerobic, motile, non-spore-forming Gram-negative rods that cause a concern in both veterinary and human medicine due to their inherent resistance and resilience against disinfectants (36). In the present study, *Non-fermenters* were identified in 28% of cases across all subgroups (36.4% in CRS), suggesting that these bacteria are not associated with any specific nasal disease (37). Repeated antibacterial courses that eliminate other commensal bacteria are likely to contribute to increased detection rates of this pathogen (11). Several studies reported the frequent detection of *Non-fermenters* in cats with CRS (6, 17, 38) and about the dilemma in treatment due to the multidrug-resistance to several antibiotics (39). A recent study showed resistance to various *β*-lactam antimicrobials with amikacin and tobramycin being the only aminoglycosides that showed consistent *in vitro* efficacy against the tested isolates (39). Culture and sensitivity testing were therefore discussed to be essential to guide therapy, as empiric antibiotic choices frequently fail to cover such resistant pathogens and the increasing prevalence of multidrug-resistant organisms highlights the importance of antimicrobial stewardship in small animal medicine (40). However, even antimicrobial treatment with reserve antibiotics targeting these multidrug-resistant bacteria in cats with CRS typically results in only temporary improvement of clinical signs (18). Despite the presence of these bacteria in cats with CRS in the present study, clinical improvement was observed following treatment with agents other than reserve antibiotics, notably doxycycline, even though *Non-fermenters* exhibit intrinsic resistance to this antibiotic (39). Additionally, some cats responded to immunosuppressants. These findings raise the question of whether other pathogens, besides *Mycoplasma* species, which are considered secondary colonizers, are detected due to the favorable environment of the “dark and wet” cavity associated with damaged epithelium in CRS patients. A similar hypothesis has been proposed in dogs with gastrointestinal diseases such as inflammatory bowel disease, where bacterial colonization is regarded as a consequence rather than the primary cause of pathology (41, 42). Likewise, in both dogs and cats with otitis externa, effective clinical improvement typically requires addressing the underlying primary disorder rather than solely targeting secondary bacterial infections (43). Nonetheless, further studies are required to substantiate this theory.

The second aim of our study focused on evaluating the usefulness of cBE in cats with CRS for guiding treatment decisions and predicting clinical outcomes. Our findings indicate that the presence of specific bacteria, whether identified by cBE or PCR, did not appear to influence prognosis or treatment response. In addition to the cBE findings, the type of nasal mucosal inflammation in cats with CRS was also not very useful in elucidating the role of bacterial colonization. However, this aspect of the study should be considered as a pilot investigation due to the small sample size and lack of reassessment through CT or endoscopy, which was only performed in two out of 11 cats.

Therapeutic options for cats with CRS mentioned in the literature include: antibiotics, antihistamines, oral decongestants (e.g., diphenhydramine hydrochloride), non-steroidal anti-inflammatory drugs, glucocorticoids, leukotriene blockers (e.g., montelukast), and immunotherapy (e.g., lipid–DNA complexes encoding the feline interleukin-2 gene) (14).

In our study, five CRS cats responded to a 21-day course of doxycycline, including two cats with bacterial isolates showing *in vitro* resistance to this antibiotic. These findings are particularly relevant in light of the rising prevalence of multidrug-resistant pathogens and the increasing use of antibiotics in CRS over extended periods. Therefore, a standardized doxycycline trial may be indicated as an initial step in a multimodal diagnostic approach, even when resistant bacteria are detected, in order to rule out other primary, secondary, or subclinical infections with organisms that are difficult to culture or with *Mycoplasma* species.

Doxycycline belongs to the group of tetracyclines, which exert a bacteriostatic effect by inhibiting protein synthesis through reversible binding to the bacterial ribosomal subunit. The antibacterial spectrum is broad, covering both gram-positive and gram-negative bacteria, including some atypical organisms like *Chlamydia* species and *Mycoplasma* species (22, 44). Longer treatment durations have been shown to be more effective in treating *M. felis* infections (22, 45). In cats with CRS, doxycycline has been administered for 21 days (46) or up to 4 to 8 weeks (3, 11, 22). Based on our findings, in contrast to acute URTD, we advise a prolonged course of doxycycline (10 mg/kg q24h or 5 mg/kg q12 hours PO for 21 days) for cats with chronic clinical signs, adjusting the treatment duration according to individual response.

Additionally, there is growing evidence that tetracyclines also modulate the immune system in various ways (47). The immunomodulatory mechanisms of action include anti-inflammatory, anti-apoptotic, and anti-proteolytic effects, as well as the inhibition of angiogenesis and tumor metastasis (48, 49). Moreover, tetracyclines inhibit matrix metalloproteinases (MMPs), which are key mediators of collagen, connective tissue, and bone destruction in various chronic inflammatory conditions (48). A recent randomized, double-blind, placebo-controlled study in humans with CRS and nasal polyposis demonstrated that doxycycline significantly improved quality of life and olfactory function (50). Of the cats in the present study responding to doxycycline, two tested negative for *Mycoplasma* species, raising the question of whether the anti-inflammatory effects of doxycycline may have contributed to clinical remission.

In kittens with acute infectious URTD, adding the antiviral medication famciclovir to topical ofloxacin for ocular manifestations and doxycycline therapy has been shown to result in statistically significant superior outcomes to those seen in kittens receiving antibiotics alone (8). Although there are data in the literature reporting improvements in cats with CRS treated with famciclovir in addition to antibiotics (51), to the authors’ knowledge, no prospective data exist on this patient group with chronic disease treated with famciclovir monotherapy. However, the two cats from the CRS Non-RD/IS group that received famciclovir following a positive FHV-1 test result showed no clinical response to treatment.

In the present study, three cats with CRS that failed to respond to doxycycline therapy showed clinical improvement to immunosuppressive therapy (oral or inhaled corticosteroids or oral cyclosporine). In accordance with clinical recommendations, viral testing had been performed before in all but one cat with CRS due to financial reasons. There is ongoing debate as to whether viral infections, particularly FHV-1, play an active role in CRS, or whether latent infections may be reactivated, especially in the context of immunosuppressive therapy (4). Positive test results for FHV-1 or FCV do not necessarily indicate the cause of clinical signs, as these viruses are also frequently detected in healthy carrier cats. Positive results may reflect the presence of vaccinal strains (11). Furthermore, it should be noted that not all cats with latent FHV-1 infection will test positive by PCR. Consequently, there is always a potential risk of viral reactivation or clinical relapse, which may lead to worsening of clinical signs. This risk was discussed with each owner prior to initiating immunosuppressive therapy. However, due to the substantial negative impact of nasal disease on the well-being of cats and the quality of life of their owners, treatment trials were pursued.

To the authors’ knowledge, a response to cyclosporine treatment in CRS cats has previously not been described, as immunosuppressive therapy with cyclosporine has been reported only in dogs with idiopathic chronic rhinitis (23). Despite limited evidence, cyclosporine was used as an alternative immunosuppressant in this study due to practical challenges associated with the administration of corticosteroids. In one cat, tapering oral steroids without concurrent inhalant therapy was not feasible due to recurrence of clinical signs, and the use of inhalants led to adverse effects (e.g., localized alopecia caused by the mask). Another cat failed to respond to corticosteroids altogether. In this context, cyclosporine was administered off-label. However, existing literature suggests that even higher dosages of cyclosporine are generally well tolerated in cats (33, 34).

The findings of this study support the hypothesis that an immune-mediated component contributes to the pathogenesis of CRS and indicate that immunosuppressive therapy could be beneficial in selected cases. Nevertheless, further studies are required to confirm these observations and establish optimal treatment protocols.

Three out of 11 cats with CRS did not show a sufficient clinical response to doxycycline or subsequent immunosuppression and were therefore classified as Non-RD/IS. These cats showed irreversible changes in the nasal cavity, such as severe turbinate destruction, which may also play a role in therapeutic response. Additionally, two of these cats tested positive for FHV-1, which may also have contributed to disease establishment and progression, despite the aforementioned limitations of interpreting FHV-1 results (11). Notably, bacteria and FHV-1 were detected in these cats, none of them experienced a worsening of clinical signs during or after immunosuppressive therapy. Further research is warranted in non-responders to determine whether superior outcomes are achieved through a combination of one or more of the following approaches: (a) targeted treatment of cultured bacteria, including oral versus topical administration; (b) anti-inflammatory or immunosuppressive therapy, as applied in the present study; or (c) antiviral therapy.

The main limitation of this clinical observational study is its retrospective design. In the present study, CRS cats were assigned to subgroups based on structured follow-up assessments conducted by ENT-experienced veterinarians. These included repeated owner interviews using validated, published questionnaires (27), as well as ongoing owner-based clinical evaluations, and a binary grading system (‘clinical signs: yes or no’) to reduce bias and improve consistency, as demonstrated in feline pain and behavior assessments (28–30). Follow-up examinations involving CT and/or endoscopy require general anesthesia and are associated with high costs and procedural risks. For this reason, and in some cases due to full clinical resolution, most owners declined anesthetized rechecks. This represents another limitation, particularly from a research standpoint, as it introduces potential bias related owner-reported outcomes. Furthermore, it is important to acknowledge the general challenge of evaluating ENT diseases in dogs and cats: clinical signs do not always accurately reflect the disease’s status (14). Nevertheless, owners were re-contacted on multiple occasions, for up to 1 year following treatment, providing insights into long-term clinical outcomes in settings of daily clinical practice.

Further limitations include the fact that cBE results may differ with regard to sample location (18) and antimicrobial pretreatment. Due to the retrospective nature of the present study, there is also a lack of standardized protocols for biochemistry, FeLV antigen and FIV antibody testing, as well as testing for FHV-1, FCV, anti-*Aspergillus* antibodies, and *Cryptococcus* antigen.

## Conclusion

The results of cBE are not diagnostic for differentiating between various nasal diseases in cats, and their diagnostic and therapeutic value in the initial work-up remains unclear. Despite *in vitro* resistance of cultured organisms, long-term clinical improvement was observed in several CRS cats treated empirically with doxycycline or immunosuppressive therapy, indicating limited correlation between cBE results and clinical outcome. This study—consistent with the literature (14)—emphasizes that cBE results should be interpreted with caution, highlighting the clinical dilemma associated with relying on cBE findings for treatment decisions. Several aspects question the routine use of cBE as it leads possibly to: (1) increased use of reserve antibiotics guided by cBE results without substantial therapeutic success; (2) potential side effects of antibiotics on feline health (e.g., disruption of the microbiome; promotion of multidrug-resistant bacteria); (3) the financial burden of cBE as part of comprehensive diagnostics in cats with nasal cavity disease, especially when PCR results for *M. felis* and other viral pathogens are available; (4) legal requirements in some countries mandating cBE prior to the prescription of certain reserve antibiotics, thereby adding to costs.

Further studies are warranted to evaluate the clinical efficacy of empirical versus guided therapy. Testing for FIV/FeLV and performing PCR for URTD-associated viruses and *Mycoplasma* species is recommended, as these findings may affect treatment approach and prognosis.

## Data Availability

The original contributions presented in the study are included in the article/supplementary material, further inquiries can be directed to the corresponding authors.
